# Noninvasive tracking of mixed venous oxygen saturation via near-infrared spectroscopy cerebral oximetry: a retrospective observational study

**DOI:** 10.1038/s41598-023-49078-1

**Published:** 2023-12-07

**Authors:** Chahyun Oh, Sujin Baek, Soomin Lee, Man-Shik Shim, Sung Joon Han, Yoon-Hee Kim, Jeong Yeon Lee, Yunseo Ku, Boohwi Hong

**Affiliations:** 1 Department of Anesthesiology and Pain Medicine, Chungnam National University Hospital, 282 Munhwa-ro, Jung-gu, Daejeon, 35015 Korea; 2 Department of Anesthesiology and Pain Medicine, Chungnam National University College of Medicine, Daejeon, Korea; 3 Department of Thoracic and Cardiovascular Surgery, Chungnam National University Hospital, Daejeon, Korea; 4 Department of Biomedical Engineering, Chungnam National University College of Medicine, Daejeon, Korea; 5 Biomedical Research Institute, Chungnam National University Hospital, Daejeon, Korea

**Keywords:** Cardiology, Medical research, Health care

## Abstract

Although previous studies have shown correlation between regional cerebral oxygen saturation (rScO_2_) and mixed venous oxygen saturation (SvO_2_), there is a lack of pragmatic information on the clinical applicability of these findings, such as tracking ability. We retrospectively analyzed continuous intraoperative recordings of rScO_2_ and SvO_2_ obtained from a pulmonary artery catheter and either of two near-infrared spectroscopy (NIRS) devices (INVOS 5100C, Medtronic; O3, Masimo) during off-pump cardiopulmonary bypass (OPCAB) surgery in adult patients. The ability of rScO_2_ to track SvO_2_ was quantitatively evaluated with 5 min interval changes transformed into relative values. The analysis included 176 h of data acquired from 48 subjects (26 and 22 subjects for INVOS and O3 dataset, respectively). The area under ROC of the left-rScO_2_ for detecting change of SvO_2_ ≥ 10% in INVOS and O3 datasets were 0.919 (95% CI 0.903–0.936) and 0.852 (95% CI 0.818–0.885). The concordance rates between the interval changes of left-rScO_2_ and SvO_2_ in INVOS and O3 datasets were 90.6% and 91.9% with 10% exclusion zone. rScO_2_ can serve as a noninvasive tool for detecting changes in SvO_2_ levels, a critical hemodynamic measurement.

## Introduction

Near-infrared spectroscopy (NIRS) is a widely used noninvasive technique in clinical practice. It was first approved by the FDA as a commercial device for measuring oxygen saturation in brain tissue and is based on the measurement of near-infrared light with a wavelength of 650–940 nm. This light can penetrate the skull and underlying cerebral tissues, allowing for the differentiation of oxyhemoglobin and deoxyhemoglobin and thereby measurement of regional oxygen saturation^1^. Unlike pulse oximetry, which mainly measures arterial blood flow, the value of regional cerebral oxygen saturation (rScO_2_) obtained through NIRS is mainly derived from venous blood, as venous blood makes up 70–75% of blood flow in brain tissue^2^.

Venous oxygen saturation, including mixed venous oxygen saturation (SvO_2_) or central venous oxygen saturation (ScvO_2_), is a critical physiological parameter that reflects the balance between oxygen supply and demand in the body. In critically ill patients, a ScvO_2_ level below 70% has been associated with poor outcomes, while a ScvO_2_ level above 90% may indicate a deteriorated oxygen extraction ratio and identify patients with a high risk of death^3^. Recently, studies have suggested transfusion strategies based on ScvO_2_ or SvO_2_ in addition to absolute hemoglobin concentration, indicating the increasing clinical utility of these parameters^4,5^. However, the invasive procedures required for these measures, such as central venous or pulmonary artery catheterization, present a significant obstacle to their clinical applicability.

As rScO_2_ is primarily determined by venous oxygen saturation, it is inevitably correlated with ScvO_2_ or SvO_2_, as shown in previous studies^6–9^. This correlation implies the potential for inferring changes in SvO_2_ noninvasively through rScO_2_. However, at present, such utilization is impeded by the lack of quantitative and practical information, including the ability to track SvO_2_ or specific thresholds for detecting significant changes. In this study, we aimed to evaluate the capability of tracking SvO_2_ by means of rScO_2_ and establish cut-off values for detecting meaningful alterations. We accomplished this by analyzing continuous intraoperative recordings obtained from a pulmonary artery catheter and two commercial NIRS devices (INVOS 5100C™, Medtronic, Mansfield, MA, USA; Root O3™, Masimo Corporation, Irvine, CA, USA) during off-pump cardiopulmonary bypass (OPCAB) surgery in adult patients.

## Results

**Figure 1:**
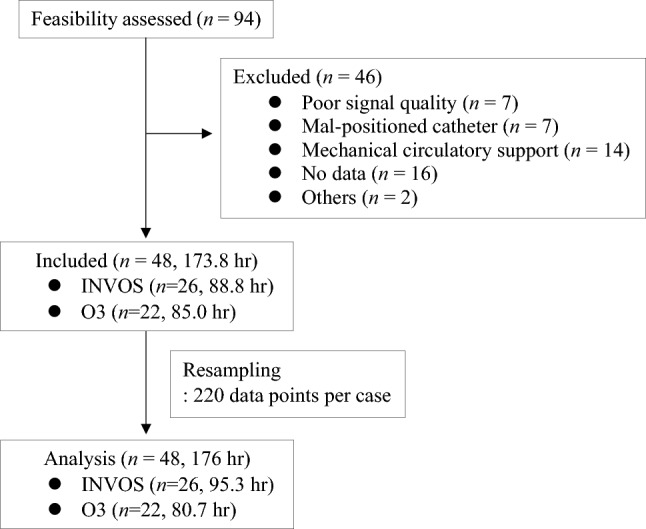
Flow diagram of patients.

A total of 94 cases were assessed for eligibility. Out of these, 46 cases were excluded from the analysis due to persistent poor signal quality (n = 7), mal-positioned catheter (n = 7), intraoperative use of mechanical circulatory assist device (n = 14), lack of available data for SvO_2_ and NIRS recordings (n = 16), insufficient rScO_2_ recording (n = 1), and unreliable SvO_2_ values (persistently over 94%, n = 1). As a result, a total of 48 subjects (with recording duration of 173.8 h) were included in the analysis, with 26 subjects in the INVOS dataset (88.8 h) and 22 subjects in the O3 dataset (85.0 h). After resampling, a total of 176 h of data were included in the analysis (Fig. 1). Baseline clinical characteristics and intraoperative measurements are summarized in Table 1.

**Table 1:** Clinical characteristics and measurements stratified by the monitoring device.

Clinical characteristics	INVOS	O3
	(n = 26)	(n = 22)
Age (yr)	66.5 ± 8.2	65.9 ± 11.0
Sex (F)	6 (23.1)	6 (27.3)
BMI (kg/m^2^)	24.1 ± 2.6	25.8 ± 4.0
Surgery type		
Minimally invasive	7 (26.9)	3 (13.6)
Conventional	19 (73.1)	19 (86.4)
Hypertension	17 (65.4)	17 (77.3)
Diabetes mellitus	16 (61.5)	14 (63.6)
Heart failure	4 (15.4)	3 (13.6)
Chronic kidney disease	4 (15.4)	5 (22.7)
Cerebrovascular disease	7 (26.9)	3 (13.6)
Left ventricular ejection fraction (< 40%)	4 (15.4)	4 (18.2)
Intraoperative inotrope/vasoactive agent use		
Vasopressin	9 (34.6)	4 (18.2)
Norepinephrine	26 (100.0)	22 (100.0)
Dobutamine	13 (50.0)	4 (18.2)
Milrinone	19 (73.1)	16 (72.7)
Duration of record (h)*	3.4 ± 1.0	3.9 ± 1.0
Mean SvO_2_ (%)	79.6 ± 6.1	80.9 ± 4.3
Mean rScO_2_ (left, %)	57.1 ± 12.7	61.7 ± 7.6
Mean rScO_2_ (right, %)	58.4 ± 13.9	63.1 ± 7.6
Mean L-R rScO_2_ difference (%)	–1.2 ± 7.3	–1.4 ± 3.3
RMSE L-R rScO_2_ difference (%)	5.9 ± 4.6	3.3 ± 1.9

Values are mean ± standard deviation or number (%).

SvO_2_, mixed venous oxygen saturation; rScO_2,_ regional cerebral oxygen saturation; L, left; R, right; RMSE, root mean square error.

*The original length of the record before resampling. After resampling, each record was equalized to 220 data points (1 min per data point).

### Correlation analysis

**Figure 2:**
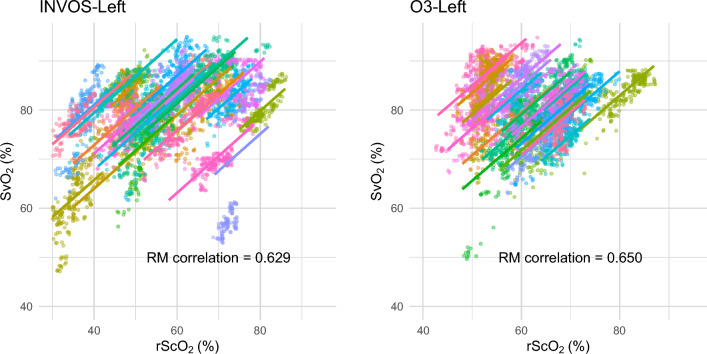
Repeated measures (RM) correlation between regional cerebral oxygen saturation (rScO_2_) and mixed venous oxygen saturation (SvO_2_). Only the left side measurements (rScO_2_) are shown. Each color represents an individual subject. Same color in both datasets (INVOS and O3) does not indicate the same subject.

Repeated measures correlation between left-rScO_2_ and SvO_2_ were 0.629 (95% CI 0.616–0.642) and 0.650 (95% CI 0.634–0.665) in INVOS and O3 datasets (Fig. 2). Repeated measures correlation between right-rScO_2_ and SvO_2_ were 0.608 (95% CI 0.592–0.62) and 0.695 (95% CI 0.676–0.712) in INVOS and O3 datasets.

### ROC curve analysis

**Figure 3:**
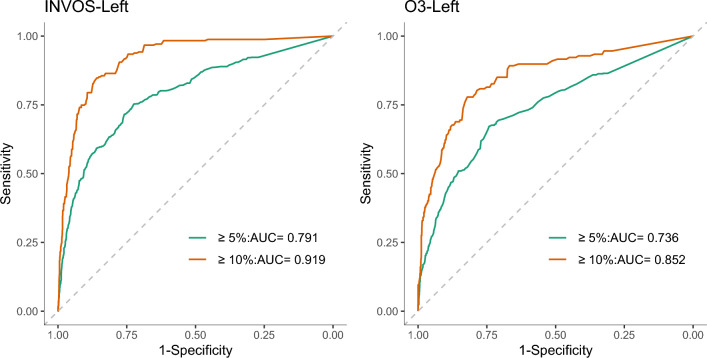
Receiver operating characteristic (ROC) curve obtained from the two datasets (INVOS and O3). Only the results of left side measurements (regional cerebral oxygen saturation, rScO_2_) are shown. The area under the curve of the ROC curve represents the performance of rScO_2_ in detecting a significant change (either 5% or 10%) in mixed venous oxygen saturation (SvO_2_) within the corresponding dataset.

Increase or decrease of SvO_2_ ≥ 5% during 5 min interval occurred 14.7% (INVOS) and 14.8% (O3) in each pooled dataset. The area under ROC (AUROC) of the left-rScO_2_ in INVOS and O3 datasets were 0.791 (95% CI 0.773–0.809) and 0.736 (95% CI 0.714–0.758). Increase or decrease of SvO_2_ ≥ 10% during 5 min interval occurred 4.2% (INVOS) and 3.5% (O3) in each pooled dataset. The AUROC of the left-rScO_2_ in INVOS and O3 datasets were 0.919 (95% CI 0.903–0.936) and 0.852 (95% CI 0.818–0.885), respectively (Fig. 3). Using a set of pragmatic cut-offs (5% and 4%), INVOS could detect ≥ 10% relative changes in SvO_2_ with sensitivity and specificity over 0.80, while O3 showed sensitivity around 0.70 and specificity over 0.80. The results of ROC curve analyses on both sides are summarized in Table 2.

**Table 2:** Performance of rScO_2_ for detecting relative changes of SvO_2_ during 5 min interval.

	INVOS						O3					
	Lt			Rt			Lt			Rt		
SvO_2_ change (%)	5	10	10	5	10	10	5	10	10	5	10	10
Cutoff (%)*	3.1	5.5	5	4.3	5.2	5	2.8	3.8	4	1.8	3.5	4
AUC	0.791	0.919	NA	0.785	0.896	NA	0.736	0.852	NA	0.751	0.860	NA
Sensitivity	0.753	0.848	0.864	0.643	0.868	0.877	0.672	0.778	0.695	0.690	0.754	0.701
Specificity	0.725	0.859	0.827	0.825	0.842	0.828	0.742	0.821	0.841	0.722	0.848	0.881
PPV	0.320	0.210	0.182	0.388	0.196	0.184	0.311	0.135	0.135	0.301	0.151	0.174
NPV	0.944	0.992	0.993	0.931	0.993	0.993	0.929	0.990	0.987	0.931	0.990	0.988
F1	0.449	0.337	0.300	0.484	0.320	0.304	0.425	0.230	0.226	0.419	0.252	0.278

*Note that the cutoffs indicate relative change of the rScO_2_ values during 5 min interval. Therefore, each cutoff can predict 5 or 10% of SvO_2_ change during 5 min interval with the corresponding performance. F1 score represents a harmonic mean of sensitivity and positive predictive value.

Lt, left; Rt, right; AUC, area under the curve; PPV, positive predictive value; NPV, negative predictive value.

### Four-quadrant plot analysis

**Figure 4:**
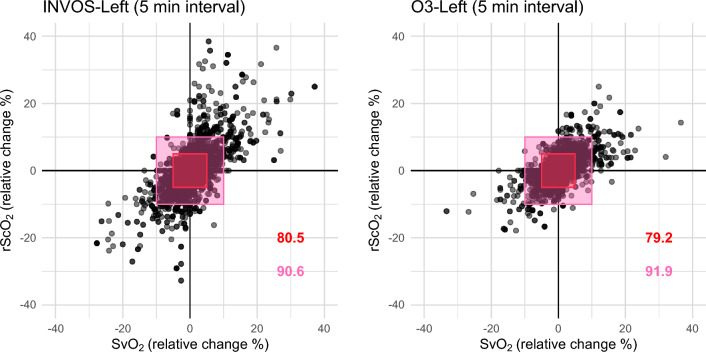
Four-quadrant plots obtained from the two datasets (INVOS and O3). Only the results of left side measurements (regional cerebral oxygen saturation, rScO_2_) are shown. Each axis indicates 5 min interval change in the corresponding parameter. The red (inner) and pink (outer) rectangles indicate exclusion zones of 5% and 10%, respectively. The concordance rates (red and pink numbers) were calculated as the proportion of points in the first and third quadrants after excluding the central exclusion zone. A concordance rate of > 92% indicates good trending ability. SvO_2_, mixed venous oxygen saturation; rScO_2_, regional cerebral oxygen saturation.

Four-quadrant plots for INVOS and O3 datasets (Left-rScO_2_) are shown in Fig. 4. The concordance rates between the interval changes of left-rScO_2_ and SvO_2_ were 80.5% and 79.2% with 5% of exclusion zone and 90.6% and 91.9% with 10% of exclusion zone in INVOS and O3 datasets. The concordance rates between the interval changes of right-rScO_2_ and SvO_2_ were 79.2% and 81.5% with 5% of exclusion zone and 86.9% and 93.1% with 10% of exclusion zone in INVOS and O3 datasets.

## Discussion

This study aimed to assess the ability of the two rScO_2_ monitoring devices in detecting changes in SvO_2_. Results indicated that these devices can be a useful tool for detecting changes in SvO_2_. The four-quadrant plot analysis demonstrated nearly acceptable trending abilities for both devices, with concordance rates of approximately 90% when using 10% exclusion zone. However, it's important to exercise caution when tracking minor degrees of SvO_2_ changes, as the capability of rScO_2_ monitoring devices to detect such small changes is limited. Based on the results, we suggest that 5% and 4% relative changes in rScO_2_ obtained from INVOS and O3 as practical thresholds for detecting 10% relative changes in SvO_2_.

Previous studies have consistently demonstrated a significant correlation between rScO_2_ and SvO_2_ (or ScvO_2_) that is considered moderate or stronger^10^. However, it is commonly acknowledged that these measurements are not interchangeable due to the bias and limited precision^11–13^. The correlation between rScO_2_ and SvO_2_ observed in our current study align with previous findings and provides additional insight into the heterogenous correlation that exists depending on the subject and the monitoring device used. As depicted in the Fig. 2, a single SvO_2_ value corresponds to a broad range of rScO_2_ values. Given the challenge of establishing an accurate model that precisely describes the relationship between these two variables for a specific subject in advance, we concur with the previous conclusions regarding the non-interchangeability of these modalities. Instead, our focus was on pragmatic trending analysis. Although our findings cannot substitute for actual SvO_2_ measurements, they do demonstrate the capability of rScO_2_ in detecting significant changes. Therefore, rScO_2_ monitoring can serve as a partial aid in decision-making regarding interventions or assessing the response to interventions.

The goal of hemodynamic management is to ensure an adequate supply of oxygen to the organs. However, determining the adequacy of oxygen delivery solely based on the amount delivered is insufficient. It's also important to consider the relation of the amount delivered to the body's requirement. In this regard, SvO_2_ plays a critical role as it reflects the balance between oxygen supply and demand. It can provide a prompt, precise, and continuous hemodynamic monitoring, making it a powerful tool for effective hemodynamic management. There is a consensus among experts, based on sound physiological principles^14^, that SvO_2_ should be continuously monitored during cardiopulmonary bypass^15^. However, outside of the field of cardiac surgery or liver transplantation, SvO_2_ monitoring is rarely practiced, mainly due to the invasive nature of the monitoring procedure, which requires pulmonary artery catheterization. Therefore, noninvasive surrogates such as rScO_2_ can be useful in clinical practice where SvO_2_ monitoring is not feasible.

The values and the interval changes of rScO_2_ and their relationship to SvO_2_ appear to exhibit discernable differences between the two devices, although the degree was not considerable. The measurements obtained from INVOS displayed a broader range of rScO_2_ values (as shown in Fig. 2), and its interval changes also covered a wider spectrum (as shown in Fig. 4). This seems to be reflected in the slightly higher cut-off value in the INVOS dataset for detecting significant changes in SvO_2_. A previous study comparing these two devices also reported characteristics that aligns with the current finding^16^. The study compared the two devices with vascular occlusion tests in healthy volunteers. Changes in tissue oxygen saturation induced by the test were more pronounced and rapid in measurements taken with INVOS compared to those with O3. Although the fundamental technology is similar, distinctions in detailed features, such as the wavelengths of near-infrared light and the computational algorithms, may account for these differences.

Careful consideration is required when interpreting and applying the results of this study, particularly in making a clear distinction between sensitivity and positive predictive value. While the study demonstrated a high sensitivity of rScO_2_ in detecting changes in SvO_2_, it should be noted that a change in rScO_2_ value does not always correspond to a significant change in SvO_2_, as indicated by relatively low positive predictive values. It is evident that deliberate or unintentional alterations in variables strongly linked to cerebral perfusion or oxygen consumption, such as carbon dioxide levels^17^ or anesthetic agents^18^, can significantly impact rScO_2_ and thereby alter the relationship between the two variables observed in this study. However, despite this limitation, true significant changes in SvO_2_ should be reflected in rScO_2_, as evidenced by the high sensitivity. It is important to recognize that the predictive value, whether positive or negative, is inevitably influenced by the incidence of the event being evaluated.

There are several limitations to this study. Firstly, due to its retrospective design, a high fidelity of the data cannot be guaranteed despite the exclusion and filtering process. Secondly, potential covariates were not strictly controlled during the measurements. As previously described, this means that the results may not hold in situations where levels of arterial oxygen saturation, carbon dioxide, or anesthesia fluctuate.

In conclusion, rScO_2_ can serve as a valuable noninvasive tool for detecting changes in SvO_2_ levels, a critical hemodynamic measurement.

## Methods

### Study design

The study was conducted in accordance with the principles of the Declaration of Helsinki. The study protocol was approved by the institutional review board of Chungnam National University Hospital (CNUH 2022–03-072), and informed consent was waived by the institutional review board. All vital data were obtained from the prospective registry of vital signs for surgical patients at Chungnam National University Hospital (CNUH 2019–08-039), which uses a free data collection program (Vital recorder^19^ version 1.8–﻿1.9, accessed at https://vitaldb.net, Seoul, Republic of Korea). The vital data registry is not targeted for a specific patient group, and the dataset used in this study is not publicly available.

This retrospective study included consecutive patients who underwent OPCAB surgery with SvO_2_ and rScO_2_ monitoring (via NIRS) from April 2021 to December 2022. Patients were excluded if their records did not include SvO_2_ and rScO_2_; if intraoperative central venous pressure wave form or postoperative chest radiography showed a mal-positioned pulmonary artery catheter; if SvO_2_ signal showed a persistent poor signal quality index (SQI = 4); or if a mechanical circulatory assist device was used. Other data collected from medical records included patient age, sex, weight, height, comorbid conditions, procedure type (conventional or minimally invasive), preoperative left ventricular ejection fraction, ant the intraoperative use of inotrope and vasoactive agents.

All patients were managed using a standardized institutional anesthetic protocol, whereby anesthesia was maintained using continuous infusion of sufentanil and rocuronium, as well as sevoflurane inhalation. Mechanical ventilation was initially set to deliver tidal volumes of 8–10 mL/kg of ideal body weight, and an inspired fraction of oxygen of 0.4–0.5, which was adjusted to maintain arterial oxygen saturation of greater than 94% and partial pressure of carbon dioxide between 35–45 mmHg.

### Data acquisition and processing

SvO_2_ data were acquired through a 7.5 F Swan-Ganz continuous cardiac output thermodilution catheter (CCOmbo V, model 774F75, Edwards Lifesciences, Irvine, CA, USA) and a HemoSphere advanced monitoring platform (Edwards Lifesciences, Irvine, CA, USA). The catheter was inserted according to the standardized institutional protocol immediately after induction of anesthesia. The catheter tip was advanced until wedging pressure was observed, withdrawn by 2–3 cm, and the balloon was deflated to prevent inadvertent wedging. Proper catheter tip placement was verified during intraoperative transesophageal echocardiography. SvO_2_ was continuously monitored following in vivo calibration during the initial stage of surgery after induction of anesthesia and stabilization of ventilatory settings and hemodynamics and recorded with 1 Hz of frequency.

Either of NIRS sensors, INVOS or O3, was chosen as the attending anesthesiologist’s preference. The NIRS sensor was then attached to the patient’s forehead before the induction of anesthesia, following the manufacturer’s guidelines. To prevent interference from ambient light and inadvertent detachments, the sensors were meticulously covered with adhesive materials. The rScO_2_ value were recorded with 0.2 and 1 Hz of frequency, respectively.

The data were extracted at a frequency of 1 Hz, and each of the two cerebral oximeter readings (left and right hemispheres) was used for analysis separately. To eliminate extreme or erroneous values, data points with SvO_2_ < 40 or > 95%, rScO_2_ < 20%, and the signal quality index of 4 (SvO_2_) were discarded. Additionally, to minimize the contribution of arterial desaturation to the tracking ability of rScO_2_, data points with SpO_2_ < 90% were discarded. Following these filtering steps, the data were averaged every 60 s and rounded to the nearest integer to match rScO_2_ and SvO_2_ values minute by minute. Finally, resampling with replacement (n = 220) was conducted to reduce potential biases stemming from unequal data lengths across subjects, with the number of resampling iterations determined based on the mean number of data points per subject.

### Statistical analysis

Based on previous studies on noninvasive estimation of central or mixed venous oxygen saturation^7,11,20^, a sample size of 20 subjects per each device was deemed sufficient for the present study. The data acquisition time frame was set to achieve this target sample size, and all feasible data were included in the analysis. Data points that had complete measurements of all three variables (left and right rScO_2_ and SvO_2_) were included in the analysis. All statistical analyses were performed using R software version 4.2.2 (R Project for Statistical Computing, Vienna, Austria).

The correlation between rScO_2_ and SvO_2_ values was assessed using repeated measures correlation analysis, which considers non-independence, using the R package ‘rmcorr’^21^. The confidence interval (CI) for repeated measures correlation was calculated using 1000 times bootstrap resampling.

To quantitatively evaluate the ability of rScO_2_ to track SvO_2_, all interval changes were transformed into relative values, before the resampling process, using a 5 min interval, as follows:

$${\text{relative}}\,{\text{change}} = ({\text{value}}_{{{\text{t}} + 5}} {-}{\text{value}}_{{\text{t}}} )/{\text{value}}_{{\text{t}}}$$

where t indicates the index time point, and t + 5 indicates 5 min after the index time point. These relative change values were then subjected to receiver operating characteristic (ROC) curve analysis and four-quadrant plot analysis^22^.

To conduct ROC curve and four-quadrant plot analyses, this study set two thresholds for significant changes (increase or decrease) in SvO_2_ values. The first threshold was based on previous research^23,24^ and set at 10% relative to the previous value, while the second threshold was set at 5% based on literature reporting a precision of 4–6% for continuous SvO_2_ monitoring via fiber-optic sensor^23,25^ and considering the mean SvO_2_ of the study cohort, which was approximately 80% (thus, 4/80 = 0.05). Youden's index was utilized to determine the optimal cut-offs for rScO_2_, with the aim of maximizing both sensitivity and specificity. The F1 score, which is a harmonic mean of precision (positive predictive value) and recall (sensitivity), was also calculated. Furthermore, based on the cut-offs determined by the Youden’s index, a set of pragmatic (simplified) cut-offs were evaluated for detecting SvO_2_ changes of ≥ 10%.

Four-quadrant plots, which involved plotting delta values of rScO_2_ and SvO_2_ on each axis, were assessed. Concordance rates were calculated by determining the proportion of data points located in the first and third quadrants, excluding the 5% and 10% exclusion zone. A concordance rate of > 92% was considered as an indication of good trending ability.

## Data Availability

The datasets generated during and/or analysed during the current study are available from the corresponding author on reasonable request.
